# Supplementary material for: Fossil bone histology reveals ancient origins for rapid juvenile growth in tetrapods
Source: Commun Biol. 2022 Nov 28;5:1280. doi: 10.1038/s42003-022-04079-0 (PMC9705711; doi:10.1038/s42003-022-04079-0)
Supplement: Supplementary file 2 — Supplementary Material [file 42003_2022_4079_MOESM2_ESM.pdf]

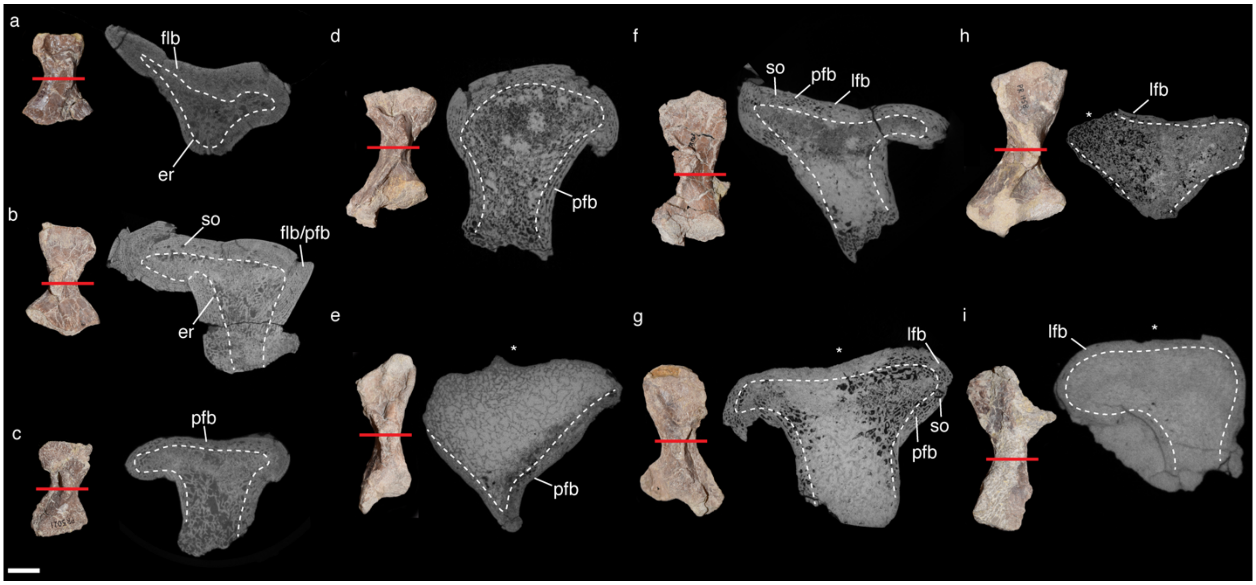

Supplementary Figure 1. μCT imagery of the entire femoral sample of *Whatcheeria deltae* in order of increasing size. Digital thin sections were collected in a transverse plane at the mid-diaphysis (red line). Dashed lines represent the endosteal surface of the cortices. All specimen photographs are in a ventral view with proximal ends to the top of the image and are to scale (scale bar = 1 cm) and all digital thin sections are oriented with the adductor crest (ventral) towards the bottom. Note: digital sections are not to scale. FMNH PR 5022 (a), FMNH PR 5021 (c), FMNH PR 1962 (f), and FMNH PR 5023 (i) were physically thin-sectioned (see Fig. 2 of main text for direct comparisons) and were used to ground-truth interpretations for additional specimens examined solely through μCT analysis. FMNH PR 1735 (b) has a thick cortex similar to (a), that is likely composed of a combination of fibrolamellar and parallel-fibred bone. FMNH PR 1952 (d) preserves a likely parallel-fibred cortex that is slightly thicker than that of FMNH PR 1992 I, although it is worth noting a considerable portion of the dorsal periosteal surface of the femur has been eroded taphonomically. Compared to FMNH PR 1962 (f), FMNH PR 1760 (g) has similar amounts of presumed parallel-fibred and lamellar-fibred tissue in the cortex. Finally, although missing a significant portion of the periosteal surface, FMNH PR 1958 (h) contains a very narrow cortex composed of lamellar bone, similar to what was observed both in μCT imagery (i) and histological thin-sectioning of FMNH PR 5023. Asterisks indicate broken or eroded edges. Abbreviations: **er**, endosteal erosion; **flb**,

fibrolamellar bone; **lfb**, lamellar-fibred bone; **pfb**, parallel-fibred bone; **so**, secondary osteon; & asterisk = eroded periosteal surface.

Supplementary Table 1. Raw values measured to determine the percent of the cross-sectional area composed of cortical bone. Values were measured in ImageJ using the histological cross-sections figured in this study.

Specimen #	Size class	Total cross-sectional area (mm^2)	Cortical bone area ($mm^{2)}$)	% cortical bone area
FMNH PR 5022	I	138.30	78.83	57%
FMNH PR 5021	II	115.33	28.83	25%
FMNH PR 1962	III	163.15	55.47	34%
FMNH PR 5023	IV	168.63	59.02	35%
