# Supplementary material for: Fossil bone histology reveals ancient origins for rapid juvenile growth in tetrapods
Source: Commun Biol. 2022 Nov 28;5:1280. doi: 10.1038/s42003-022-04079-0 (PMC9705711; doi:10.1038/s42003-022-04079-0)
Supplement: Supplementary file 3 — Reporting summary [file 42003_2022_4079_MOESM3_ESM.pdf]

nature portfolio

Corresponding author(s): Megan Whitney

Last updated by author(s): Sep 20, 2022

Reporting Summary

Nature Portfolio wishes to improve the reproducibility of the work that we publish. This form provides structure for consistency and transparency in reporting. For further information on Nature Portfolio policies, see our Editorial Policies and the Editorial Policy Checklist.

Statistics

For all statistical analyses, confirm that the following items are present in the figure legend, table legend, main text, or Methods section.

n/a	Confirmed	
☐	☒	The exact sample size (*n*) for each experimental group/condition, given as a discrete number and unit of measurement
☐	☒	A statement on whether measurements were taken from distinct samples or whether the same sample was measured repeatedly
☒	☐	The statistical test(s) used AND whether they are one- or two-sided<br>*Only common tests should be described solely by name; describe more complex techniques in the Methods section.*
☒	☐	A description of all covariates tested
☒	☐	A description of any assumptions or corrections, such as tests of normality and adjustment for multiple comparisons
☒	☐	A full description of the statistical parameters including central tendency (e.g. means) or other basic estimates (e.g. regression coefficient) AND variation (e.g. standard deviation) or associated estimates of uncertainty (e.g. confidence intervals)
☒	☐	For null hypothesis testing, the test statistic (e.g. *F*, *t*, *r*) with confidence intervals, effect sizes, degrees of freedom and *P* value noted<br>*Give P values as exact values whenever suitable.*
☒	☐	For Bayesian analysis, information on the choice of priors and Markov chain Monte Carlo settings
☒	☐	For hierarchical and complex designs, identification of the appropriate level for tests and full reporting of outcomes
☒	☐	Estimates of effect sizes (e.g. Cohen's *d*, Pearson's *r*), indicating how they were calculated

Our web collection on statistics for biologists contains articles on many of the points above.

Software and code

Policy information about availability of computer code

Data collection	ImageJ
Data analysis	No software was used

For manuscripts utilizing custom algorithms or software that are central to the research but not yet described in published literature, software must be made available to editors and reviewers. We strongly encourage code deposition in a community repository (e.g. GitHub). See the Nature Portfolio guidelines for submitting code & software for further information.

Data

Policy information about availability of data

All manuscripts must include a data availability statement. This statement should provide the following information, where applicable:

- Accession codes, unique identifiers, or web links for publicly available datasets
- A description of any restrictions on data availability
- For clinical datasets or third party data, please ensure that the statement adheres to our policy

All histological images have been uploaded as single, high-resolution images to MorphoBank for widespread availability of the data presented here. Access to these images can be found in MorphoBank under Project P4272:Ontogenetic histology of Whatcheeria deltae. Images will be release upon publication.

Human research participants

Policy information about studies involving human research participants and Sex and Gender in Research.

Reporting on sex and gender	N/A
Population characteristics	N/A
Recruitment	N/A
Ethics oversight	N/A

Note that full information on the approval of the study protocol must also be provided in the manuscript.

Field-specific reporting

Please select the one below that is the best fit for your research. If you are not sure, read the appropriate sections before making your selection.

☐ Life sciences ☐ Behavioural & social sciences ☒ Ecological, evolutionary & environmental sciences

For a reference copy of the document with all sections, see nature.com/documents/nr-reporting-summary-flat.pdf

Ecological, evolutionary & environmental sciences study design

All studies must disclose on these points even when the disclosure is negative.

Study description	Nine femora from a range of size classes were either thin-sectioned or micro-CT scanned for this project. Sections were taken from the mid-shaft of the femora to detail the growth record of the nine different individuals.
Research sample	Femora from Whatcheeria were identified from collections by experts in the taxonomy and anatomy of this animal.
Sampling strategy	We sampled as many identifiable specimens as possible from each of the previously four recognized size classes for Whatcheeria. Destructive thin-sectioning was done on one specimen per size class and confirmation of these observations were obtained from additional sampling using non-destructive micro-CT scanning.
Data collection	Specimens were identified from previous collections. All specimens were photographed, CT scanned, casted and molded for preservation prior to destructive sampling. Histological data was obtained following standard paleohistological methodologies.
Timing and spatial scale	The specimens sampled here are from the same quarry
Data exclusions	No data were excluded
Reproducibility	All histological images are available for independent interpretation
Randomization	The sampling for this study is based on fossil collections from a single quarry.
Blinding	This was not an experimental study but rather a sampling of the fossil record. That said, the author responsible for describing the histology was unaware of the size classes (determined by a co-author) prior to analysis.

Did the study involve field work? ☐ Yes ☒ No

Reporting for specific materials, systems and methods

We require information from authors about some types of materials, experimental systems and methods used in many studies. Here, indicate whether each material, system or method listed is relevant to your study. If you are not sure if a list item applies to your research, read the appropriate section before selecting a response.

Materials & experimental systems

n/a	Involved in the study
☒	☐ Antibodies
☒	☐ Eukaryotic cell lines
☐	☒ Palaeontology and archaeology
☒	☐ Animals and other organisms
☒	☐ Clinical data
☒	☐ Dual use research of concern

Methods

n/a	Involved in the study
☒	☐ ChIP-seq
☒	☐ Flow cytometry
☒	☐ MRI-based neuroimaging

Palaeontology and Archaeology

Specimen provenance

No new specimens were collected for this study. All specimens come from a quarry on Jasper Heimstra's private property and collections were made during 1985, 1986, and 1988 and therefore permits were not required. Permission from the land owner to collect on their land was provided. Since the collection of the specimens, they have been accessioned at the Field Museum of Natural History.

Specimen deposition

Field Museum of Natural History

Dating methods

No new dates are provided.

☐ Tick this box to confirm that the raw and calibrated dates are available in the paper or in Supplementary Information.

Ethics oversight

No ethical requirements or oversight were required for these specimens as they were collected by sanctioned museum field teams and are not species or sights that require such oversight.

Note that full information on the approval of the study protocol must also be provided in the manuscript.
